# Improving access to cancer clinical research in Brazil: recent advances and new opportunities. Expert opinions from the 4th CURA meeting, São Paulo, 2023

**DOI:** 10.3332/ecancer.2024.1698

**Published:** 2024-04-18

**Authors:** Heloisa Resende, Roberto J Arai, Carlos H Barrios, Fernanda Schwyter, Nelson L S Teich, Andre Gomes, Analluza B Dallari, Laís A S Bonilha, Claudiosvam M A Souza, Fernando R Francisco, Rodrigo R Munhoz, Gustavo Werutsky, Marisa Madi, Paulo Fernandes, Jose M Figueiredo, Fabio Fedozzi, Lilian Arruda, Vinícius Q Aguiar, Andreia C Melo

**Affiliations:** 1 Instituto Projeto Cura, São Paulo 05507-020, SP, Brazil; 2 Independent Clinical Researcher, São Paulo 01153-000, SP, Brazil; 3 Latin American Cooperative Oncology Group (LACOG), Porto Alegre 90619-900, RS, Brazil; 4 Instituto Teich de Políticas Públicas em Saúde, Rio de Janeiro 22640-102, RJ, Brazil; 5 Bristol Myers Squibb, São Paulo 04719-002, SP, Brazil; 6 Independent Lawyer, São Paulo 01153-000, SP, Brazil; 7 Comissão Nacional de Ética em Pesquisa (CONEP), Brasília 70719-040, DF, Brazil; 8 Agência Nacional de Vigilância Sanitária – Anvisa, Brasília 71205-050, DF, Brazil; 9 Associação Brasileira de Organizações Representativas de Pesquisa Clínica- Abracro, São Paulo 01311-902, SP, Brazil; 10 Hospital Sírio Libanês, São Paulo 01308-050, SP, Brazil; 11 Sociedade Brasileira de Oncologia Clínica (SBOC), São Paulo 01311-300, SP, Brazil; 12 Sail for Health, São Paulo 04543-011, SP, Brazil; 13 Instituto do Câncer Brasil, Taubaté 12030-200, SP, Brazil; 14 Associação Brasileira de Linfoma e Leucemia, ABRALE, São Paulo 05423-040, SP, Brazil; 15 Hospital São Camilo, São Paulo 05022-001, SP, Brazil; 16 Centro Universitário de Volta Redonda – UNIFOA, Volta Redonda 27240-560, RJ, Brazil; 17 Brazilian National Cancer Institute (INCA), Division of Clinical Research and Technological Development, Rio de Janeiro 20230-130, RJ, Brazil; a https://orcid.org/0000-0003-4692-3743; b https://orcid.org/0000-0001-5617-1042; c https://orcid.org/0000-0001-6021-667X; d https://orcid.org/0000-0003-1352-1334; e https://orcid.org/0000-0001-8898-2798; f https://orcid.org/0000-0001-6271-105X; g https://orcid.org/0000-0003-1049-4932; h https://orcid.org/0000-0002-7101-4325; i https://orcid.org/0000-0002-6257-0119; j https://orcid.org/0000-0002-1201-4333

**Keywords:** cancer, clinical trials, low- and middle-income countries

## Abstract

Clinical research is the cornerstone of improvements in cancer care. However, it has been conducted predominantly in high-income countries with few clinical trials available in Brazil and other low-and-middle-income countries (LMIC). Of note, less than one-third of registered clinical trials addressing some of the most commonly diagnosed cancers (breast, lung and cervical) recruited patients from LMIC in the last years. The Institute Project CURA promoted the fourth CURA meeting, discussing barriers to cancer clinical research and proposing potential solutions. A meeting was held in São Paulo, Brazil, in June 2023 with representatives from different sectors: Brazilian Health Regulatory Agency (Anvisa), National Commission of Ethics in Research (CONEP), non-governmental organisations, such as the Latin American Cooperative Oncology Group, the Brazilian Society of Clinical Oncology (SBOC), Contract Research Organisations, pharmaceutical companies and investigators. A total of 16 experts pointed out achievements as shortening the time of regulatory processes involving Anvisa and CONEP, development of staff training programs, maintenance of the National Program of Oncological Attention (PRONON), and the foundation of qualified centres in North and Northeast Brazilian regions. Participants also highlighted the need to be more competitive in the field, which requires optimising ongoing policies and implementing new strategies as decentralisation of clinical research centres, public awareness campaigns, community-centered approaches, collaborations and partnerships, expansion of physicians-directed policies, exploring the role of the steering committee. Active and consistent reporting of the initiatives might help to propagate ongoing advances, increasing Brazilian participation in clinical cancer research. Engagement of all players is crucial to maintain continuous progress with further improvements in critical points including regulatory timelines and increments in qualified human resources which aligned with new educational initiatives focused on physicians and the general population will expand access to cancer clinical trials in Brazil.

## The worldwide imbalance between burden cancer and cancer clinical trials distribution: how is Brazil positioned?

Clinical and epidemiological research has been the cornerstone of improvements in cancer care [1], and it has been predominantly conducted in high-income countries (HIC), with few studies conducted in Latin America (LATAM) and other low- and-middle-income countries (LMIC) [2,3]. Of note, less than one-third of registered clinical trials addressing some of the most commonly diagnosed cancers (breast, lung and cervical) recruited patients from LMIC in 2010–2017 [4]. This imbalance regarding cancer clinical trial distribution, aligns poorly with the global burden of cancer which widens disparities in cancer care by concentrating cancer knowledge generation, application, and infrastructure within HICs [5]. Also, there is a recent recommendation by the American Society of Clinical Oncology (ASCO) and the Association of Community Cancer Centers that a commitment across research stakeholders is necessary to increase equity, diversity and inclusion, and clinical trials are an integral component of high-quality cancer care, so every person with cancer should have the opportunity to participate [6].

Brazil is the LATAM country with the highest participation in cancer clinical research, however, it is far lower than expected. Brazil is positioned worldwide as the seventh pharmaceutical market and sixth in population [7], having ethnic diversity [8] and a high number of cancer cases [9]. All of these features should bring more opportunities for cancer clinical research participation. Promoting major access to clinical trials is vital to optimising cancer care [10], modifying clinical practice, bringing more treatment opportunities, increasing qualified human resources, and ultimately generating academic research initiatives that can bring secondary gains by conducting trials that certainly will address local relevant questions [11]. Therefore, the diagnosis of the different barriers to cancer clinical trial access in Brazil is crucial.

One important issue, regarding the current scenario is a long approval process which is partially explained by the relatively recent participation of LATAM countries in global clinical trials. From 1996 when the Brazilian resolution CNS 196/96 came into force [12], conceptual and structural grounds of ethics regulation in Brazil have been consistently implemented. Parallelly, the Brazilian Health Regulatory Agency (Anvisa) has obtained international recognition for its work and became an International Conference of Harmonisation member in 2016 [13], and manager member in 2021. Since their creation, the National Commission of Ethics in Research (CONEP) and Anvisa have worked to improve regulatory approvals in Brazil, but timelines remain longer than timelines in HIC, requiring continued efforts.

Other barriers that hamper the major Brazilian participation in cancer clinical research are the paucity of clinical trials available, centralisation of research centres with adequate infrastructure in the capitals and big cities, low engagement of physicians, lack of qualified human resources, scarcity of clinical research awareness by patients and general population. Of note, to modify several points in this landscape, efforts by society, government, policymakers, non-governmental institutions, investigators, and pharmaceutical companies are required.

The CURA Project Institute, a nonprofit organisation, established in 2016, is one of few institutions in LATAM whose objectives are to raise attention among the general population on the importance of clinical research. CURA also promotes scientific events aiming to foster the Brazilian regulatory environment, encourage health professionals to become researchers and create a philanthropic culture in favour of academic research for the control and cure of cancer. Since 2021, the Institute has promoted the ‘CURA meetings’, which have brought the opportunity to gather some players of regulatory processes, assistance, pharmaceutical companies, and medical societies to discuss the current scenario and suggest potential solutions.

The fourth CURA meeting was held in São Paulo in June 2023, setting together several experts in the field. During this meeting, three speakers presented the up-to-date situation concerning cancer clinical research in Brazil followed by a discussion with the experts from different sectors, highlighting the main barriers, pointing out the achievements in recent years and suggesting strategies to face the present challenges. The information provided by the experts was used to build the narrative below.

## Recent advances in cancer clinical research scenario in Brazil

**Figure 1.**
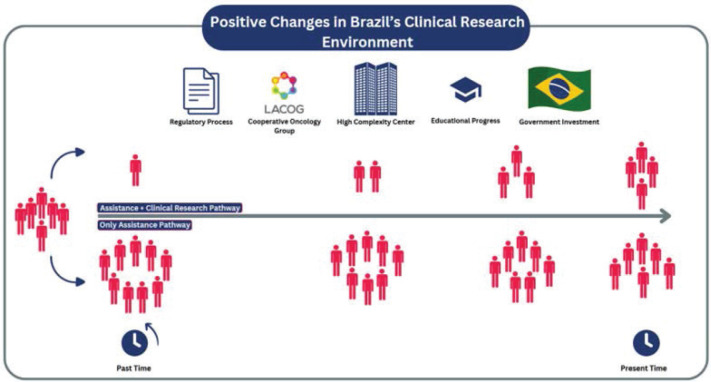
Positive changes in Brazil’s clinical research environment.

Currently, Brazil ranks 20th worldwide in cancer clinical research [7], and the meeting participants agreed that the country is moving forward concerning opportunities, participation, and positive aspects in the national scenario (Figure 1). Of note, this meeting represented a milestone in cancer clinical research in Brazil as it brought players from different sectors, including Anvisa and CONEP, pharmaceutical companies, non-governmental organisations representing patients, Brazilian Society of Clinical Oncology (SBOC), Latin America Cooperative Oncology Group (LACOG) and investigators, covering rich discussion focused on barriers in each specific sector.

The timeline of changes in the clinical research environment in Brazil points to determining factors that have altered the flow of patients from only assistance pathway to assistance plus clinical research pathway. These factors include advances in regulatory processes, the participation of cooperative oncology group, the expansion of high-complexity centres, progress in research education and government investments in the area*.*

### Regulatory approvals

The last decade’s adjustments in the regulatory processes, involving Anvisa and CONEP, have allowed Brazil to become more competitive. Anvisa has conducted its actions within a strategic plan aiming to align regulatory processes with best international practices, achieving greater predictability and shortening the timelines. Some of these actions are incorporating collaborative practices in the regulatory process through the Collegiate Board Resolution *Resolução da Diretoria Colegiada* (RDC) 741 published on 10 August 2022 [14], which established general rules. Reliance is a practice endorsed by the World Health Organisation which permits one national regulatory authority to consider previous evaluations by regulatory authorities from other countries (*Autoridades Regulatórias Estrangeiras Equivalentes*) intending to substantiate its own decisions [15]. Such procedure has optimised internal practices, allowing shortening of evaluation times. The clinical research department within Anvisa elaborated two specific Resolutions of the Collegiate Board of Directors (RCDs), 573 in 2021 (573/2021) and 601 in 2022 (601/2022) [16, 17] both documenting reliance applied to clinical research. Regarding complex or exception clinical trials, represented by national development of products, biologic products, and phase I and II trials, the RDC 573/2021 established a maximum period of 120 days for Anvisa to manifest. Regarding clinical trials not classified as an exception, RDC 601/2022 does not establish a maximum period for Anvisa manifestation but specifies simplified and optimised rules according to reliance which has allowed a shorter approval time.

Improvements have also been noticed regarding ethical approvals involving the Institutional Review Board (IRB) in Portuguese *Comitê de Ética em Pesquisa* (CEP) and CONEP, known as the CEP-CONEP system, which nowadays is based on a triple protocol analysis, involving the nominated coordinator IRB, CONEP and each participating site local IRB [18]. CONEP preconizes the need for approval by the coordinator and local IRB, as well as CONEP, as a strategy to ensure the rights of the participants. However, CONEP agrees that for expediting these approval processes periodic training programs for IRBs must be adopted [19], and in addition the accreditation of new IRBs is mandatory [20]. Despite the fact the current approval times are long even compared to other LATAM countries, they are smaller than 2 years ago [21, 22].

### Cooperative oncology groups

The creation of Academic Cancer Research Groups is a recognised strategy to increase participation in clinical research [23]. The LACOG is a multicentre collaborative cancer group launched in 2009, with most members from Brazil, but also from other LATAM countries, and a coordinating office located in Porto Alegre, Brazil [24]. LACOG has presented expressive growth in the last years, of note, a rise of scientific publications of around 95% in the last decade. LACOG has assisted investigators in the study concept, protocol development and management, monitoring, data management, pharmacovigilance, statistical analysis and the publication of the results. Also, LACOG has developed its own research projects which are sponsored by pharmaceutical companies and diverse grants, including governmental. Presently, LACOG manages tumour groups (breast, gastrointestinal, genitourinary, geriatric, gynaecological, head and neck, lung, neuro, radiation, sarcoma and digital health) responsible for educational and retrospective, translational or clinical research initiatives [25].

Reinforcing the role of staff training, LACOG has qualified investigators, nurses, and study coordinators for 14 years now, since its creation [25]. These persons are distributed in cancer research centres across Brazil.

### High-quality centres

Brazil has many cancer research sites with adequate infrastructure and personnel [22] which have received Food and Drug Administration (FDA) inspections and pharmaceutical companies auditing with no major findings [26]. However, they are usually associated with universities, institutes, or academic groups in cities such as São Paulo, Rio de Janeiro, and Porto Alegre. For instance, the *Pontifícia Universidade Católica do Rio Grande do Sul* research centre in Porto Alegre, has conducted cancer clinical research for 20 years [27], with more than 300 studies, 2,500 included patients [28] denoting that Brazil already has a long history with meaningful achievements. The COVID-19 pandemic has also demonstrated the local potential in clinical research development. The world health crisis shed light on Brazil´s role in the vaccine research and clinical trials enterprise. The widespread contagion, a deep bench of active scientists, and a manufacturing infrastructure have made Brazil an important player in the tracking to find a vaccine [29, 30].

The number of sponsored and non-sponsored trials increased substantially in response to the need for solutions to control the pandemic. In line with urgent requirements, regulatory agencies readjusted processes to speed up protocol analysis. So, regardless of all the research limitations, Brazil has trained investigators who can conduct and propose clinical research with some qualified centres to support the initiatives. It was a demonstration that Brazilian investigators and centres are able to develop high-quality work in research that can prosper with the correct incentives.

### Educational-oncologist-centred programs

One barrier that inhibits major Brazilian participation in clinical trials is the low engagement of physicians [31], highlighting the need for educational-oncologist-centred programs. The SBOC has around 3,000 members. According to the Medical Demography 2023 [32], Brazil has 4,730 physicians registered at the Federal Council of Medicine somehow working in the field of clinical oncology (including not only the medical oncologists by training). Therefore, SBOC has a representative number of members in the field and represents an important space for training in different topics, including clinical research in oncology. Among training actions are the creation of the Society Clinical Research Committee [33] which plays an active role in preparing the scientific program for the research section at the SBOC Annual Congress and the development of a training program for oncologists in cancer clinical research. Initially conducted in Ijuí (a middle size city located in the state of *Rio Grande do Sul*), which is outside the Rio-São Paulo axis and has an outstanding active research centre, currently, this program has many other involved centres. The program is focused on oncologists working outside large centres intending to implement a Clinical Research Center in their region and consists of an immersion period at a high-quality research centre. Following the training steps the oncologists are connected to a clinical oncology research network and receive support in their initial projects. In the beginning, only three oncologists were trained per year. As of 2022, the training centres have been expanded and currently, 15 oncologists have been trained yearly.

Recently, SBOC created an annual research fund *Fundo de Incentivo à Pesquisa* (FIP) that aims to finance research projects with resources from the SBOC focusing on reducing disparities and seeking equal access to cancer treatment [34]. The grant will be allocated to projects approved by a judging committee, composed of members appointed by the SBOC. The SBOC hopes to motivate Brazilian researchers working with cancer, including young oncologists, to create research projects that can address local issues.

### Governmental investments

The Brazilian government’s policy to support clinical research is still incipient, however, there are few programs that have brought great opportunities. One of the most important strategies is an online platform called ‘*Plataforma Brasil*’ [35] which has modified the regulatory environment once it is used for the entire process of ethics appraisal. This platform brought traceability, organisation, and speed to the registered protocols in Brazil.

Another worthy government program, which is also a source of funding is the National Program of Oncological Attention *Programa Nacional de Apoio à Atenção Oncológica* (PRONON), created in 2014 [36] and recently renewed [37], representing an opportunity for investigators and cooperative groups to propose and conduct academic research. A great example of how PRONON can foster clinical research initiatives in oncology is the NEOSAMBA project [38]. It is academic research with the expenses covered by PRONON, that investigates the better sequencing of two chemotherapy protocols used in neoadjuvant treatment in breast cancer. In summary, PRONON helps cancer patients by answering relevant questions, promoting more clinical research opportunities, and training and retaining human resources in Brazilian research centres.

## New opportunities to improve cancer research scenario in Brazil

**Figure 2.**
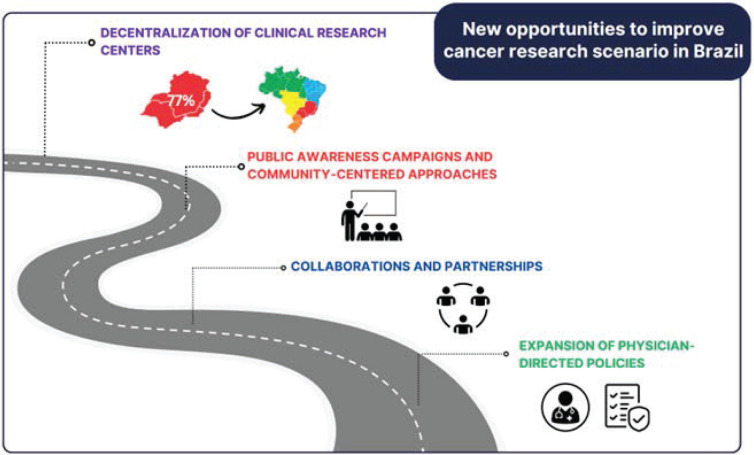
New opportunities to improve cancer research scenario in Brazil.

Even though many achievements have been made, there is an agreement that Brazil needs improvement in many other points (Figure 2) in addition to the maintenance of the continued efforts in topics that already have been conquered. Also, there was a consensus about the complexity of this landscape and the necessity of the involvement of all players through this journey (Table 1).

The road to improving the current scenario of clinical cancer research in Brazil is based on the need to improve current policies and implement new strategies as the decentralisation of clinical research centres, public awareness campaigns, community-centred approaches, new collaborations and partnerships and the expansion of physician-directed policies.

### Decentralisation of clinical research centres

The concentration of clinical research centres in high-developed regions in Brazil is only an additional topic of the major centralisation of health care services, which hinders accessibility [39]. Accessibility is an important factor associated with variations in the use of health systems, thus poor geographic accessibility to healthcare services contributes to low utilisation, which in turn gives rise to poorer health outcomes [40]. In Brazil, 77% of the clinical research centres are located in the South and Southwest regions [22]. The qualification of new centres in North, Northwest and Central-West regions, aiming for decentralisation of clinical research which is concentrated in South and Southwest regions and capital cities, is an urgent action. LACOG has worked for 2 years in partnership with *Instituto Vencer o Câncer* [41, 42], training teams in new research centres. In 2023, six new sites in North and Northwest regions received support, and three of them have ongoing studies. The next efforts will be applied to maintain the qualification programs as new sites need careful attention on the staff capacitation and infrastructure once the learning curve may be long [43, 44]. Notably, the partnering may engage sponsors to prioritize actions such as capacitation programs for new research sites in regions where no clinical trials are found.

The principal investigator with a consistent clinical research background is critical to lead new sites. Therefore, institutions should cooperate with initiatives of this type together with sponsors or study promoters that can allocate studies according to the infrastructure availability, complexity, and patient population. It is expected that new sites require extra oversight from all parties. This includes an experienced clinical research associate to monitor and advise the sites, a proficient study manager to handle risks and extra support from the institution.

The main challenge is to expand the number of centres throughout Brazil to increase the density of protocols per habitant and organise training programs with sites to initiate low-complexity studies, with high-quality services that in the future can manage more complex studies.

### Public awareness campaigns and community-centred approaches

Participation in clinical trials in oncology worldwide has remained low, between 2% and 8% of adults with cancer, although most of the patients in cancer clinical trials report favourable experiences [6]. One important reason is overly restrictive eligibility criteria, which have been revisited by the ASCO and FDA, trying to modernise and broad inclusion criteria that will permit more generalisability of data [45]. However, this low accrual represents a multifactorial scenario including, local culture, resource barriers, misperception regarding clinical research, and lack of interest by patients and physicians [46]. Participants of clinical trials tend to be younger, healthier and represent a less diverse population in terms of race, ethnicity and geographical distribution than people in daily clinical practice [47]. Raising awareness among the general public concerning the importance of clinical research and its potential benefits can foster a culture of research participation. Increasing community engagement represents a strategy to face this complex landscape and facilitate the recruitment of participants by dispelling misconceptions and fears surrounding clinical trials. Utmost, in an engaged community, more individuals may be encouraged to volunteer for studies. Also, it can target marginalised groups given widespread distrust stemming from long-standing racism and discrimination which also ensures diversity among participants. The community-centred strategies promote the dissemination of clinical trials, raising transparency across cancer research. Funding agencies are increasingly recognising the importance of community engagement in the research process, and it has become a benchmark for large research programs funded by the National Institutes of Health [48]. LACOG has worked together with CURA to promote actions having a focus on the community [49]. These actions include, for instance, educational meetings, workshops, and lives for the naïve audience regarding clinical research. Recently, LACOG and CURA are convinced of the need to create a new platform where the potential participants will be able to find clinical research opportunities across the country, with friendly and clear language.

### Collaborations and partnerships

Strengthening collaborations and partnerships will be necessary for increasing Brazilian participation in cancer research programs. Facilitating alliances among stakeholders such as academic and private institutions, pharmaceutical companies, and international organisations can promote knowledge exchange and innovation in the clinical research field. On the other hand, partnering between those stakeholders and patient-representative organisations to prioritise common objectives is crucial. Regarding collaboration among stakeholders, it would be meritorious for instance, for qualified and knowledgeable centres to offer training and support for new centres in their initial studies, pharmaceutical companies sponsoring training programs to new centres offering within these programs the opportunity to qualify for the first clinical trial, centres collaborating one each other by conducting academic studies, partnering public–private centres which would permit using the structure of both. The first step may be to connect stakeholders to raise awareness of each one about their roles.

### Expansion of physicians-directed policies

Physicians have enormous importance on patient enrolment in clinical trials, however, several barriers have hindered the referrals. Of note, a recent Brazilian survey described that one-third of the oncologists refer only 1% of their patients to clinical trials [31]. In opposite, a meta-analysis evaluating clinical trial participation in the United States described that 55% of invited patients accepted to participate, standing out that patients are willing to take part in the clinical research as long as they are invited [50]. Among reasons that might explain this low rate of referral by Brazilian physicians are the paucity of available clinical trials, the lack of a unified and updated platform managed by a reliable institution with available trials, the need for referral patients to distant centres, competing patient care demands in public hospitals with scarce resources, clinician biases which make them judge the patient unwilling or unable to comply with trial protocols [51]. These barriers may be particularly acute at hospitals where oncologists are not affiliated with research networks. Once again, LACOG has developed expressive engagement of oncologists qualifying and giving the opportunity of new centres to get their first participation in academic or low-complexity-pharma-sponsored trials. The number of available studies will gather and motivate new oncologists, ultimately modifying the rate of referral in the country. Creating more opportunities for participation of new centres certainly will bring more training programs, which are necessary to improve physicians’ capacity to enrol patients. Many studies demonstrated that initial communication with patients is highly variable, and many researchers lack training in how to talk with potential participants about clinical trials [52].

Another crucial issue related to physicians is the remuneration model. The wages of the investigators are usually inadequate in LATAM countries, leading to the preference for clinical practice rather than a career in clinical research [53]. The medical doctors who work on clinical research activities and maintain clinical practice are often overwhelmed with clinical duties and are not provided with adequate protected time for conducting research [2]. Thus, policies clarifying the need for an appropriate remuneration model in clinical research probably will attract more physicians.

## Conclusion

Brazil has presented expressive gains in regulatory processes and educational strategies, standing out leadership by the LACOG and the SBOC. Also, some governmental initiatives regarding research funding have fostered academic research. However, continued efforts are necessary, mainly to become Brazil more competitive which requires shorter and more predictable timelines, unified, updated and free access clinical trials platform, continuing educational program physician-directed and public awareness campaigns. The landscape is excessively complex and needs further engagement of all policymakers, pharmaceutical companies, investigators, cooperative groups, medical societies, non-governmental organisations, government and general society to transform it into a more inclusive scenario.

## Conflicts of interest

Heloisa Resende has received research funding from Novartis and Roche, all outside the scope of this manuscript.

Gustavo Werutsky has received research funding from AstraZeneca/MedImmune, Bristol-Myers Squibb Brazil, Pfizer, Roche and Roche/Genentech, all outside the scope of this manuscript. Has consulting or advisory role at Merck.

Carlos H Barrios has received research funding from AB Science, Abbvie, Abraxis BioScience, Amgen, Asana Biosciences, Astellas Pharma, AstraZeneca, Biomarin, Boehringer Ingelheim, Bristol-Myers Squibb, Celgene, Clinica Atlantis, Covance, Daiichi Sankyo, Exelixis, GlaxoSmith-Kline, Halozyme, ImClone Systems, INC Research, inVentiv Health, Janssen, LEO Pharma, Lilly, Medivation, Merck, Merck KGaA, Merrimack, Millennium, Mylan, Novartis, Pfizer, PharmaMar, Polyphor, Roche/Genentech, Sanofi, Shanghai Henlius Biotech and Taiho Pharmaceutical, all outside the scope of this manuscript. Has consulting or advisory role at AstraZeneca, Boehringer Ingelheim, Eisai, GlaxoSmithKline, Libbs, Lilly, MSD Oncology, Novartis, Pfizer, Roche/Genentech and United Medical. Has stock and other ownership interests in MedSIR and Tummi.

All other authors have no conflicts of interest to disclose.

## Funding

None.

## Figures and Tables

**Table 1.** Clinical research landscape in Brazil.

Recent advances		New opportunities	
What has been done	How it has improved the scenario	What else should be done	How it would improve the scenario
Working on regulatory approvals	Reliance adoption	Decentralisation of clinical research centres	To bring new centres close to patients
	Shortening timelines		To increase the density of protocols per habitant
Creation of cooperative oncology groups	Provide investigators support	Public awareness campaigns and community-centred approaches	Raising awareness among the public concerning the importance of CR
	Management of tumour groups		Dispelling misconceptions and fears surrounding CR
	Staff training		Raising representativity in CR
Presence of high-quality centres and educational-oncologist-centred programs	Qualified centres with notable participation in CR	Collaborations and partnerships	Qualified centres could offer training and support for new centres in their initial studies
	Expressive role during COVID pandemic		Centres could collaborate one each other by conducting academic studies
Disponible government investments	*Plataforma Brasil*	Expansion of physicians-directed policies	Widen training programs
	PRONON		Time-protected for PI working on CR

CR: Clinical Research; PI: Principal Investigator; PRONON: Programa Nacional de Apoio à Atenção Oncológica
